# Interstitial Lung Disease in Patients With Unresectable Stage III NSCLC Treated With Chemoradiotherapy Followed by Durvalumab in Japan: Analysis From the Multicenter Prospective AYAME Study

**DOI:** 10.1111/1759-7714.70299

**Published:** 2026-05-19

**Authors:** Nobuyuki Yamamoto, Hirotsugu Kenmotsu, Kiichiro Ninomiya, Burak Akdemir, Shinya Uematsu, Ayako Fukui, Ryo Koto, Masakazu Fujiwara, Chikako Iwao, Hiroshi Kitagawa, Ichiro Yoshino, Akihiko Gemma, Tetsuya Mitsudomi, Yoshinobu Saito

**Affiliations:** ^1^ Internal Medicine III Wakayama Medical University Wakayama Japan; ^2^ Division of Thoracic Oncology Shizuoka Cancer Center Shizuoka Japan; ^3^ Center for Comprehensive Genomic Medicine Okayama University Hospital Okayama Japan; ^4^ Oncology Medical, AstraZeneca K.K Osaka Japan; ^5^ Evidence and Observational Research, Medical, AstraZeneca K.K Osaka Japan; ^6^ Department of Thoracic Surgery International University of Health and Welfare Narita Hospital Chiba Japan; ^7^ Department of Pulmonary Medicine and Oncology, Graduate School of Medicine Nippon Medical School Tokyo Japan; ^8^ Division of Thoracic Surgery, Department of Surgery Kindai University Faculty of Medicine Osaka Japan; ^9^ Department of Pulmonary Medicine Nippon Medical School Musashi Kosugi Hospital Kanagawa Japan

**Keywords:** carcinoma, chemoradiotherapy, durvalumab, interstitial, lung diseases, non‐small‐cell lung

## Abstract

**Introduction:**

Interstitial lung disease (ILD) is a concerning adverse event associated with immune checkpoint inhibitors, including durvalumab. This analysis of the multicenter AYAME study assessed the longterm safety of durvalumab, focusing on ILD.

**Methods:**

AYAME enrolled patients prescribed durvalumab for unresectable stage III non‐small cell lung cancer (NSCLC) after chemoradiotherapy (CRT) from July 2019 to December 2020 in Japan. Patients received durvalumab for ≤ 12 months and were prospectively followed for 3 years. Incidence, severity, and management of ILD were examined. Multivariable logistic regression analysis was conducted to investigate the association of patient characteristics with grade ≥ 2 ILD occurrence.

**Results:**

Of 511 patients in the safety analysis population, ILD occurred in 383 patients (75.0%) from durvalumab initiation to subsequent treatment start; median time to occurrence was 44.0 days. ILD led to permanent durvalumab discontinuation in 121/383 patients (31.6%), dose interruption in 111/383 (29.0%), and corticosteroid intervention in 168/383 (43.9%). Grade ≥ 2 ILD occurrence was higher in patients with volume of lung parenchyma that received 20 Gy (V20) ≥ 25% versus < 25% (60.3% vs. 29.4%; adjusted odds ratio 2.20, 95% confidence interval 1.08–4.48), 5 Gy (V5) ≥ median (37.85%) versus < median (51.5% vs. 27.2%; 1.79, 1.07–3.00), and grade 1 ILD before durvalumab administration versus no ILD (50.0% vs. 37.6%; 2.11, 1.07–4.17).

**Conclusions:**

This study of durvalumab for NSCLC after CRT in a real‐world setting indicated factors associated with grade ≥ 2 ILD and provided valuable insights into ILD management strategies. These findings may contribute to establishing effective approaches to minimize ILD risk in practice.

**Trial Registration:** UMIN000037090/NCT03995875

## Graphical abstract

Interstitial lung disease associated with chemoradiotherapy followed by durvalumab in unresectable stage III NSCLC: findings from the prospective multicenter AYAME study.

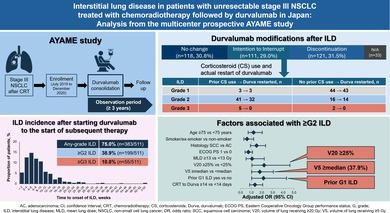

## 1 Introduction

Lung cancer remains the leading cause of cancer deaths worldwide [1], including in Japan [2]. Non‐small cell lung cancer (NSCLC) accounts for 80%–90% of lung cancers [3], and about one‐third of NSCLC cases have stage III disease at the time of diagnosis [4].

In the PACIFIC trial, patients receiving durvalumab, a high‐affinity programmed death‐ligand 1 (PD‐L1) inhibitor, for unresectable stage III NSCLC after chemoradiotherapy (CRT), had significantly prolonged progression‐free survival (PFS) and overall survival (OS) compared with those receiving placebo [5, 6, 7]. Consolidation therapy with durvalumab after CRT (the PACIFIC regimen) has become the standard‐of‐care for unresectable stage III NSCLC [5, 6, 7], and is recommended in this setting in lung cancer clinical practice guidelines in Japan, Europe, and the United States [8, 9]. However, few large‐scale studies had prospectively evaluated the long‐term effectiveness and safety of durvalumab, including subsequent treatment. To address this knowledge gap, we conducted a real‐world study (the AYAME study) that assessed the safety and effectiveness of durvalumab after CRT in Japanese patients with unresectable stage III NSCLC [10]. Patients were prospectively followed for 3 years, including during the post‐durvalumab treatment period. The study demonstrated comparable effectiveness to other studies, with a median real‐world PFS (rwPFS) of 23.2 months and a 3‐year OS rate of 65.6% [10].

Immune‐mediated adverse events, including interstitial lung disease (ILD), may occur for a long time after completion or discontinuation of immune checkpoint inhibitors [11]. The frequency of ILD during the durvalumab treatment period was 74.4% in the AYAME study [10], which was similar to that observed in Japanese patients in the PACIFIC trial (73.6%) [12], but higher than the overall PACIFIC trial population (33.9%) [5]. However, more detailed information is needed to optimize the clinical management of this clinically‐relevant adverse event, including patient characteristics associated with ILD occurrence, appropriate intervention for ILD, and its incidence after treatment completion. Several ILD‐related values in this manuscript differ from those reported in the previous publication of the AYAME study [10], because that analysis included ILD events that occurred after the initiation of subsequent treatment; events during that period were not included in the present analysis.

The aim of this analysis of the AYAME study was to evaluate the long‐term safety of durvalumab, with a focus on ILD, to specifically learn about Japanese patients with stage III unresectable NSCLC treated with the PACIFIC regimen.

## 2 Methods

### 2.1 Study Design

AYAME was a multicenter, noninterventional cohort study (UMIN000037090; NCT03995875) that enrolled patients prescribed durvalumab for unresectable stage III NSCLC after CRT from July 2019 to December 2020 at 52 sites in Japan [10]. Patients received durvalumab for a maximum of 12 months and were prospectively followed for 3 years from the start of durvalumab treatment until the date of discontinuation, death, or final survival, as applicable. Patient follow‐up was completed in December 2023. Patient demographic and clinical data were collected using electronic case report forms.

The study was performed in accordance with the ethical principles of the Declaration of Helsinki and the Japanese ethical guidelines for medical and biological research involving human subjects. The final study protocol, informed consent forms, protocol amendments, and advertisements for recruitment were approved by the Ethics Committee at each site.

### 2.2 Patients

Patients with unresectable stage III NSCLC planning to initiate durvalumab treatment after CRT, and who provided written informed consent, were enrolled. The details of CRT were not specifically defined. Patients with recurrence after surgical resection of the primary lesion were eligible, provided that they had N2 or N3 unresectable stage III NSCLC. The study excluded patients aged < 20 years and those who had participated in any interventional clinical study using unapproved drugs or off‐label use of drugs from the first diagnosis to the end of durvalumab treatment.

### 2.3 Outcomes

The main outcome of this analysis of the AYAME study was to assess the incidence of ILD that occurred between the start date of durvalumab treatment and the date of the first subsequent treatment, discontinuation, death, or final survival, whichever was the earliest (Main Analysis Period). Investigators were provided with a predefined list of ILD event terms including “radiation pneumonitis,” “alveolitis,” “interstitial lung disease,” “pneumonitis,” “acute interstitial pneumonitis,” “diffuse alveolar damage,” and “pulmonary fibrosis” for prospective event collection. ILD events identified through radiological imaging were assessed and diagnosed by attending physicians.

The incidence of ILD after the start of subsequent therapy (Subsequent Period) was also evaluated. The medications used for subsequent treatment were categorized into epidermal growth factor receptor tyrosine kinase inhibitors (EGFR‐TKI), other TKI, immune‐oncology (IO) drugs, chemotherapy ± other, and IO drugs plus chemotherapy ± other. “Other” was defined as any drugs other than EGFR‐TKI, other TKI, and IO (i.e., anti‐angiogenic agents).

### 2.4 Statistical Analysis

The safety analysis population comprised all enrolled patients who received at least one dose of durvalumab. Descriptive statistics were used to summarize categorical variables with frequencies and proportions and continuous variables with number of observations, medians, and ranges (minimums, maximums). ILDs were summarized with the number and proportion of patients and events and for patient subgroups.

A multivariable logistic regression analysis was conducted to determine the relationship between patient characteristics and the incidence of grade ≥ 2 or grade ≥ 3 ILD during the Main Analysis Period. Adjusted odds ratio (aOR) and corresponding 95% confidence intervals (CIs) were estimated and adjusted for several prespecified patient characteristic covariates (age, smoking status, histology, Eastern Cooperative Oncology Group performance status [ECOG PS] before durvalumab initiation, mean lung dose [MLD] of irradiation, volume of lung parenchyma that received 20 Gy of irradiation [V20], volume of lung parenchyma that received 5 Gy of irradiation [V5], ILD status before the start of durvalumab treatment, and time from the end of CRT to the start of durvalumab), and forest plots were created. The explanatory variables were pre‐specified and were determined from what is clinically relevant to ILD.

Subgroup analyses were also conducted to evaluate ILD incidence according to radiation parameters (e.g., V20 and V5 categories), irradiation methods (intensity‐modulated radiation therapy [IMRT] vs. three‐dimensional conformal radiation therapy [3D‐CRT]), and subsequent treatment types following durvalumab.

Missing data were handled as “missing” when analyzing continuous variables or summarizing data by category, and not imputed but included in the denominator when calculating proportions and summary statistics. For the categorization of continuous variables, the cutoff values were prespecified: the median was used as the cutoff for V5, while the cutoffs for V20 and MLD were based on the publication by Murakami and colleagues [13].

Planned interim analyses were performed annually between 2021 and 2023. The results of these analyses have been previously reported at conferences and did not lead to a change in the study design; the final analysis data are reported herein.

All analyses were performed using SAS 9.4 or more updated versions (Cary, NC, USA).

## 3 Results

### 3.1 Patient Disposition and Baseline Characteristics

A total of 529 patients were enrolled, of whom 512 were initiated on durvalumab: two patients withdrew consent, seven did not meet the eligibility criteria, and eight were excluded for other reasons (Figure S1). The safety analysis population comprised 511 of these 512 patients; one patient was excluded because the informed consent obtained was not considered appropriate.

Patients in the safety analysis population had a median age of 69.0 years and 119 (23.3%) were aged ≥ 75 years (Table S1). Most patients (76.9%) were male, 13.9% were current smokers, 74.4% were former smokers, and most had an ECOG PS of 0 (51.3%) or 1 (46.8%). A total of 210 patients (41.1%) in the safety analysis population completed the 12‐month durvalumab treatment period.

### 3.2 ILD During Main Analysis Period

**FIGURE 1:**
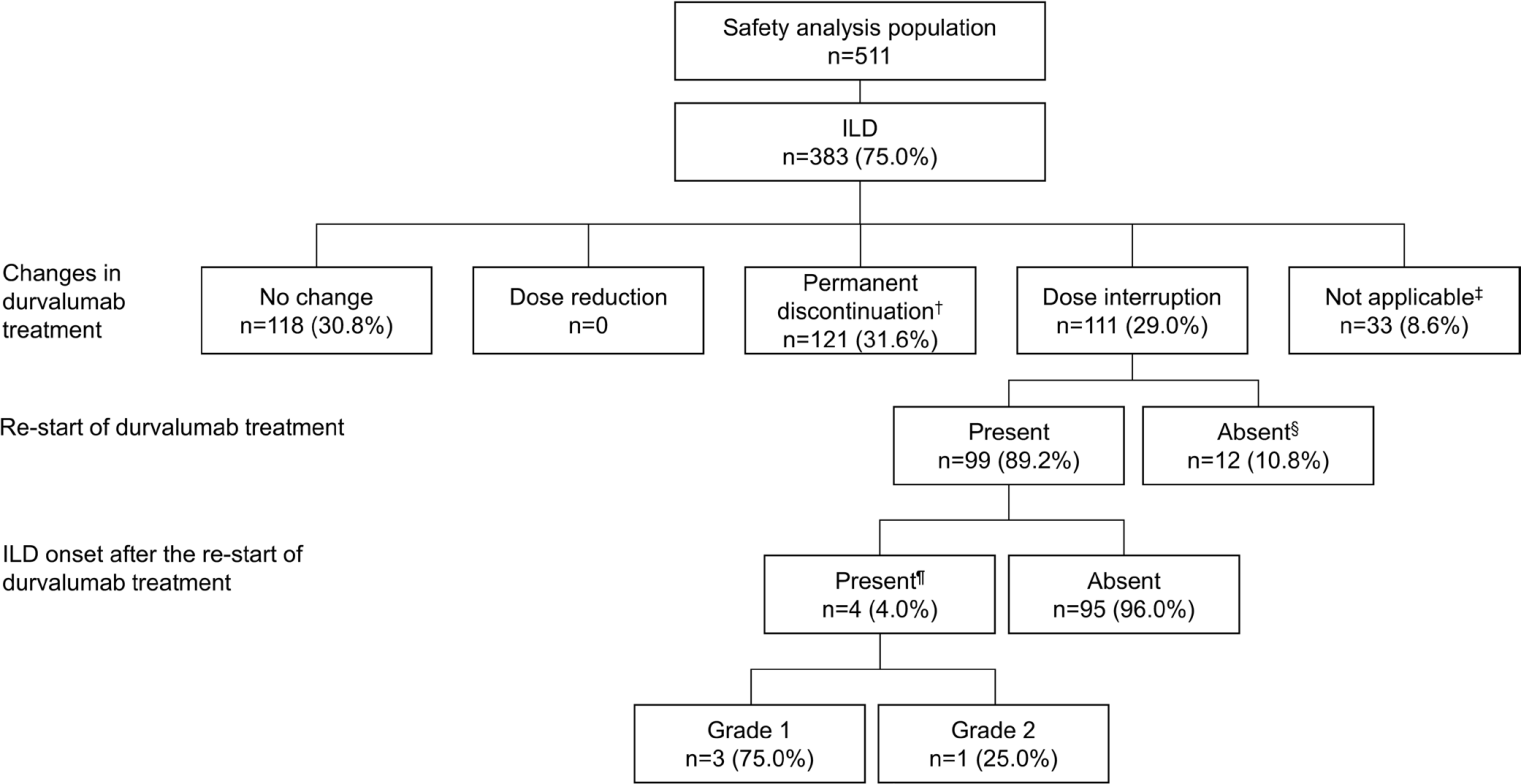
Intervention for ILD that occurred during the main analysis period. ILD events were counted from the start of durvalumab treatment until the initiation of subsequent treatment. ^†^Cases where treatment was discontinued without dose interruption and cases where treatment was interrupted before being permanently discontinued. ^‡^Cases where durvalumab was not the suspected drug and cases where durvalumab was the suspected drug but had already been discontinued before ILD onset. ^§^Cases where treatment was discontinued for reasons other than ILD (e.g., disease progression). ^¶^Only those cases that were counted as new‐onset. ILD, interstitial lung disease.

In the safety analysis population (*n* = 511), ILD was reported in 383 patients (75.0%) during the Main Analysis Period, from the start of durvalumab treatment until the initiation of subsequent treatment (Figure 1, Table S2); of these, the ILD was considered grade 1 (*n* = 184, 36.0%), grade 2 (*n* = 144, 28.2%), grade 3 (*n* = 51, 10.0%), grade 4 (*n* = 0), and grade 5 (*n* = 4, 0.8%) in severity. The median time to first onset of ILD following the first dose of durvalumab was 44.0 days. ILD onset led to permanent discontinuation of durvalumab in 121/383 patients (31.6%) and dose interruption in 111/383 (29.0%).

Corticosteroid intervention for ILD was required by 168/383 patients (43.9%; Table 1). The use of corticosteroids was associated with ILD severity; corticosteroids were administered to eight of 184 (4.3%) patients with grade 1, 110 of 145 (75.9%) with grade 2, and 50 of 54 (92.6%) with grade ≥ 3 ILD.

**TABLE 1:** Intervention for ILD categorized by presence or absence of intervention with corticosteroid therapy in patients who developed ILD in the safety analysis population.

Maximum ILD grade	Intervention with corticosteroid therapy (*n* = 383)	
	Presence (*n* = 168)	Absence (*n* = 215)
	*n* (%)	*n* (%)
1	8 (4.8)	176 (81.9)
2	110 (65.5)	35 (16.3)
3	46 (27.4)	4 (1.9)
4	0	0
5	4 (2.4)	0

*Note:* ILD events were counted from the start of durvalumab treatment until the initiation of subsequent treatment.

Abbreviation: ILD, interstitial lung disease.

Change in durvalumab treatment by ILD grade is shown in Table S3. Of the 118 patients who did not require any change in durvalumab treatment following ILD occurrence, most had grade 1 ILD (109/118 patients, 92.4%). Of the 121 patients who permanently discontinued durvalumab due to ILD, most had grade 2 (63/121, 52.1%) or grade 3 (38/121, 31.4%) ILD, followed by grade 1 ILD (16/121, 13.2%). Among the 111 patients who required durvalumab dose interruption, the majority had grade 2 (57/111, 51.4%) or grade 1 (47/111, 42.3%) ILD, with a smaller proportion experiencing grade 3 ILD (7/111, 6.3%).

Of the 111 patients who experienced dose interruption for ILD onset, 99 were able to restart durvalumab treatment, while the remaining 12 patients could not resume treatment due to disease progression or other reasons. Among the 99 patients who restarted durvalumab, corticosteroid intervention was required in 40 patients (40.4%; Table S4). Four patients (4.0%) experienced new episodes of ILD after re‐starting durvalumab (grade 1, *n* = 3; grade 2, *n* = 1; Figure 1). None of these patients required corticosteroid treatment.

**FIGURE 2:**
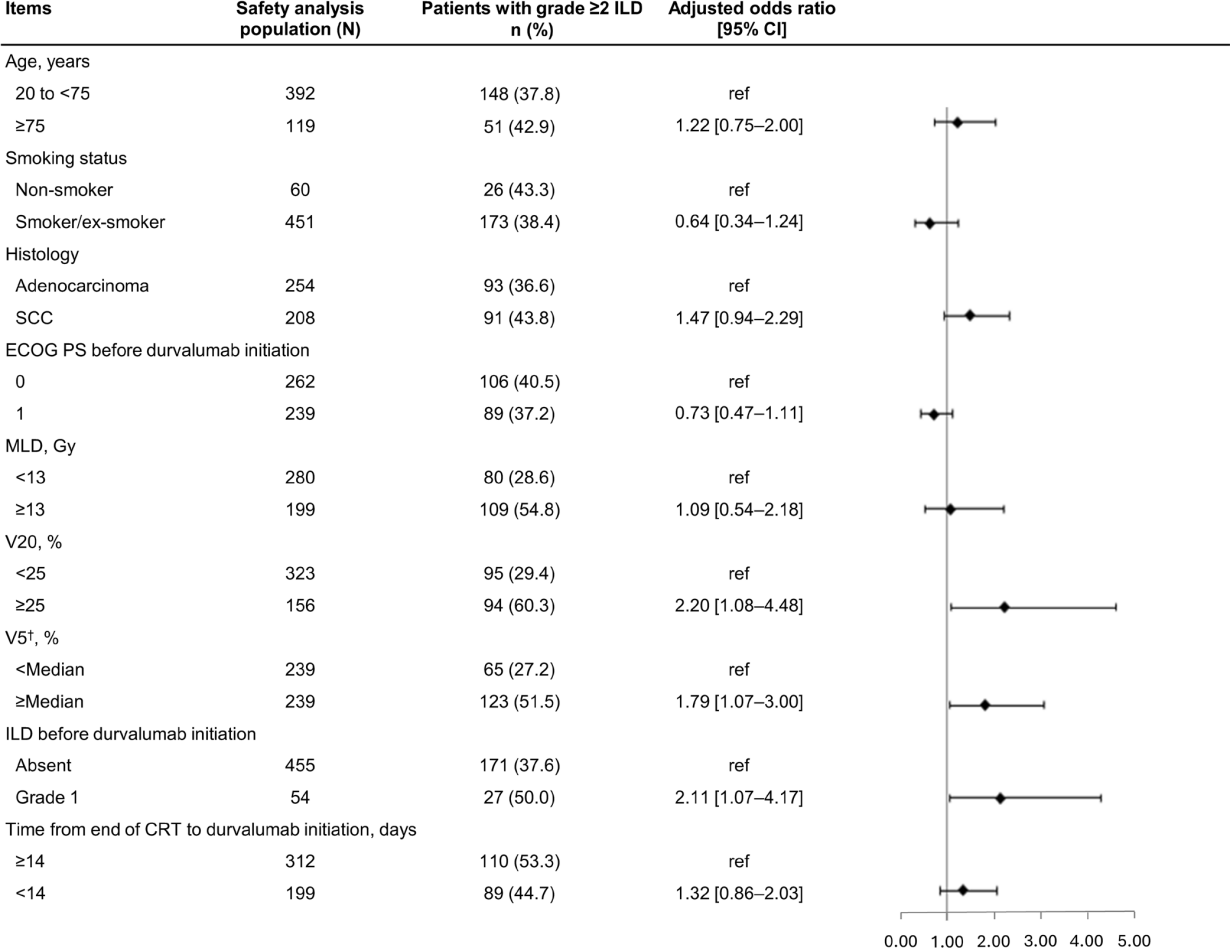
Multivariable logistic regression analysis of grade ≥ 2 ILD occurrence in the safety analysis population. ILD events were counted from the start of durvalumab treatment until the initiation of subsequent treatment. ^†^Median = 37.85%. CI, confidence interval; CRT, chemoradiation therapy; ECOG PS, Eastern Cooperative Oncology Group performance status; ILD, interstitial lung disease; MLD, mean lung dose; SCC, squamous cell carcinoma; V20, volume of lung parenchyma that received 20 Gy; V5, volume of lung parenchyma that received 5 Gy.

**FIGURE 3:**
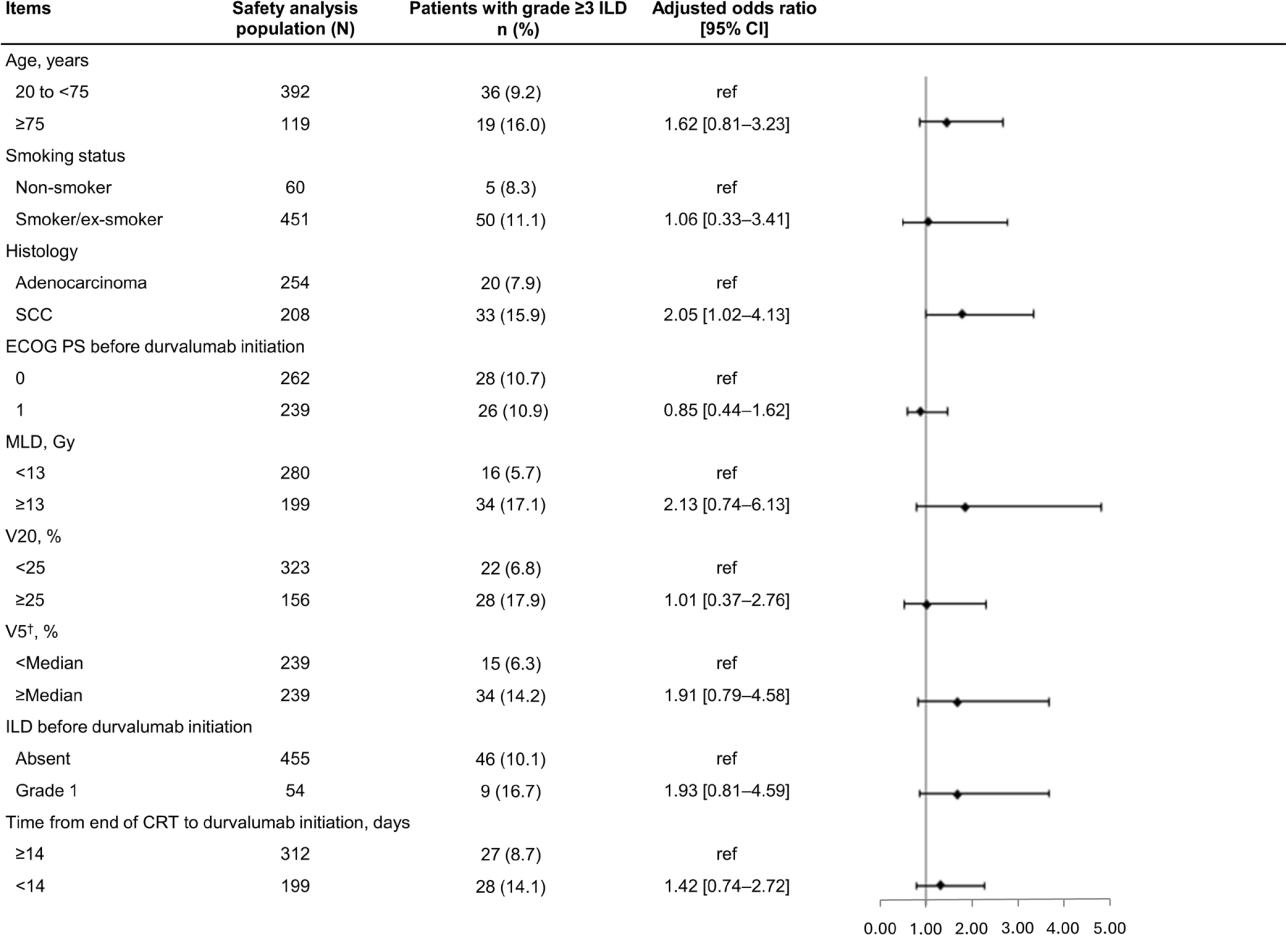
Multivariable logistic regression analysis of grade ≥ 3 ILD occurrence in the safety analysis population. ILD events were counted from the start of durvalumab treatment until the initiation of subsequent treatment. ^†^Median = 37.85%. CI, confidence interval; CRT, chemoradiation therapy; ECOG PS, Eastern Cooperative Oncology Group performance status; ILD, interstitial lung disease; MLD, mean lung dose; SCC, squamous cell carcinoma; V20, volume of lung parenchyma that received 20 Gy; V5, volume of lung parenchyma that received 5 Gy.

Multivariable logistic regression analysis showed that the risk of grade ≥ 2 ILD occurrence was higher in patients with V20 ≥ 25% versus < 25% (60.3% vs. 29.4%; aOR 2.20, 95% CI 1.08–4.48), V5 ≥ median (37.85%) versus < median (51.5% vs. 27.2%; aOR 1.79, 95% CI 1.07–3.00), and grade 1 ILD before durvalumab administration versus no ILD (50.0% vs. 37.6%; aOR 2.11, 95% CI 1.07–4.17; Figure 2). Further, multivariable logistic regression analysis showed that the risk of grade ≥ 3 ILD occurrence was higher in patients with squamous cell carcinoma histology versus adenocarcinoma (15.9% vs. 7.9%; aOR 2.05, 95% CI 1.02–4.13; Figure 3).

In the subgroup analysis of ILD categorized by V20, of 323 patients with V20 < 25%, the incidence of any grade ILD and grade ≥ 3 ILD was 70.6% and 6.8%, respectively, which was numerically lower than in the 156 patients with V20 ≥ 25% to < 30%, ≥ 30% to < 35%, or ≥ 35% (86.1%–93.5% and 14.9%–33.3%, respectively; Table S5). Likewise, in 239 patients with V5 < median (37.85%), the incidence of any grade ILD and grade ≥ 3 ILD were 66.5% and 6.3%, respectively, which was numerically lower than the corresponding rates in the 239 patients with V5 ≥ median (86.2% and 14.3%, respectively; Table S6).

Regarding the irradiation method, the incidence of ILD in patients treated with IMRT (*n* = 160) was comparable with that observed in the 358 patients who received 3D‐CRT (any grade 76.3% vs. 73.5%, respectively, and grade ≥ 3 10.6% vs. 12.3%, respectively; Table S7).

### 3.3 ILD During Subsequent Period

In the analysis of ILD after the start of subsequent treatment, the incidence of ILD was higher when subsequent treatment regimens included IO alone (6/20, 30.0%) compared with EGFR‐TKI (1/23, 4.3%), chemotherapy/other (9/121, 7.4%), other TKI (1/9, 11.1%), or IO plus chemotherapy/other (4/32, 12.5%; Table 2). Onset of ILD usually occurred within 34 weeks from the start of subsequent treatment administration (Figure S2). Of the 23 patients who received first‐line subsequent EGFR‐TKI therapy, 17 received EGFR‐TKI therapy more than 6 months after the last administration of durvalumab (data not shown). Of these 17 patients, one (5.9%) developed ILD, which was categorized as grade 1.

**TABLE 2:** ILD after the start of first subsequent treatment by first subsequent treatment regimen in the safety analysis population.

	First subsequent treatment regimen				
	EGFR‐TKI^a^ (*n* = 23)	Other TKI^b^ (*n* = 9)	IO alone^c^ (*n* = 20)	Chemo/other^d^ (*n* = 121)	IO + chemo/other^e^ (*n* = 32)
Events^f^, *n* (%)	1 (4.3)	1 (11.1)	6 (30.0)	9 (7.4)	4 (12.5)
ILD grade					
1	1 (100.0)	0	2 (33.3)	3 (33.3)	1 (25.0)
2	0	0	4 (66.7)	0	2 (50.0)
3	0	1 (100.0)	0	3 (33.3)	0
4	0	0	0	0	0
5	0	0	0	2 (22.2)	1 (25.0)
Unknown	0	0	0	1 (11.1)	0

Abbreviations: Chemo, chemotherapy; EGFR‐TKI, epidermal growth factor receptor‐tyrosine kinase inhibitor; ILD, interstitial lung disease; IO, immune‐oncology; TKI, tyrosine kinase inhibitor.

^a^ EGFR‐TKI: gefitinib, erlotinib, afatinib, osimertinib, and dacomitinib.

^b^ Other TKI: crizotinib, alectinib.

^c^ IO: nivolumab ± ipilimumab, pembrolizumab, atezolizumab.

^d^ Chemo (cytotoxic anticancer drugs)/other: any drugs other than those listed as EGFR‐TKI, other TKI, and IO.

^e^ IO + chemo/other: a combination of drugs listed as IO and chemo/other.

^f^ Patients with > 1 event were counted for each event (duplicate counting).

## 4 Discussion

This comprehensive analysis provided detailed insights into associated factors and management of ILD in Japanese patients, while the AYAME study [10] had reported the overall incidence of ILD in patients treated with the PACIFIC regimen. Our findings indicated factors associated with grade ≥ 2 ILD, including V20 ≥ 25%, V5 ≥ median (37.85%), and presence of grade 1 ILD before durvalumab administration. Furthermore, this analysis provided valuable clinical insights regarding post‐ILD management strategies, outcomes following re‐administration of durvalumab after discontinuation, and ILD occurrence during subsequent therapies.

During the Main Analysis Period (from the initiation of durvalumab treatment until the start of subsequent therapy), ILD occurred in 75.0% of patients, of whom 43.9% required corticosteroid intervention. The incidence of ILD was comparable with that reported in previous Japanese real‐world retrospective studies (81.0%–83.1%) [14, 15], as well as Japanese patients in the PACIFIC trial (73.6%) [12], but higher than the rate observed in the real‐world PACIFIC‐R study (17.9%) involving patients from Australia and Europe [16]. The proportion of patients with ILD who received corticosteroid intervention in our analysis (43.9%) was higher than that reported in one of the previous Japanese real‐world studies (25%) [15], which may be related to the higher proportion of grade ≥ 2 ILD observed in our cohort.

In our analysis, the majority of patients who continued durvalumab treatment without any modification had grade 1 ILD, whereas most patients who required some form of treatment modification had grade ≥ 2 ILD. Following on from dose interruption (as a result of ILD), durvalumab was re‐started in 89.2% of patients, with four patients (4.0%) subsequently experiencing a new episode of ILD (grade 1, *n* = 3; grade 2, *n* = 1). We suggest that appropriate management, including corticosteroid administration for grade ≥ 2 ILD in accordance with the Japanese Pharmaceutical and Medical Devices Agency report on the deliberation results for durvalumab [12], might be the reason for the low rate of ILD recurrence in our study.

In a multivariable analysis, V20 ≥ 25% was considered to be a factor that may be associated with the occurrence of grade ≥ 2 ILD (aOR 2.20), which was similar to that reported in an observational, cohort study in Japan (OR 2.37) [15]. Similarly, V5 ≥ median (37.85%) might be associated with grade ≥ 2 ILD occurrence; a previous report supports this finding [17]. IMRT is known to expand the low‐dose irradiation area which could lead to increased V5 [17]. In our study, the incidence of ILD with IMRT was comparable with that seen with 3D‐CRT. This suggests that the irradiation modality itself is not associated with the incidence of ILD, and that management of V5 is also essential. Regarding the occurrence of grade ≥ 3 ILD, multivariable analysis showed that squamous cell carcinoma may be a factor associated with its occurrence. This finding is consistent with a previous study that also suggested squamous cell carcinoma as a factor for the occurrence of severe ILD [18]. Given that smoking burden is strongly associated with the development of squamous cell lung cancer [19], this histological type may reflect a higher smoking exposure among these patients compared with smokers with other histological types.

The incidence of ILD in the Subsequent Period was not particularly high when IO drugs were not used for subsequent treatment. More than half of the first‐line subsequent treatment regimens consisted of cytotoxic anticancer drugs, which is similar to a previous report [20]. In our study, the incidence of any grade ILD with subsequent EGFR‐TKI treatment after durvalumab was 4.3%, which was lower than in a previous study of patients who received subsequent EGFR‐TKI treatment after PD‐(L)1 inhibitors, where grade ≥ 3 ILD was observed in 4/41 patients (9.8%) [21]. In that study, all four patients who experienced grade ≥ 3 ILD had received nivolumab or pembrolizumab and most patients started EGFR‐TKI within 3 months from immunotherapy [21]. In our study, no cases of ILD were observed within 3 months following the completion of durvalumab treatment; however, it is important to note that only three of the 23 patients who received subsequent EGFR‐TKI were treated within 3 months of durvalumab completion. The longer interval between durvalumab completion and subsequent EGFR‐TKI treatment in our study may be associated with the lower rate of ILD. Of note, the incidence of ILD in our study was highest in patients receiving first‐line subsequent treatment regimens that included IO alone (30.0%) compared with other regimens (4.3%–12.5%). Therefore, when selecting a treatment that includes IO after the PACIFIC regimen, the risk of ILD should be considered.

This study had several limitations. First, since the AYAME study was conducted in Japan, the findings from this analysis, such as risk factors for ILD, may not be generalizable to populations worldwide. For example, Asian patients undergoing CRT with or without immunotherapy appear to have a higher incidence of radiation pneumonitis than their non‐Asian counterparts [22]. This is thought to be because of genetic differences between the two populations, as well as the possibility that Asian patients receive higher radiation doses to the lung and exhibit reduced pulmonary tolerance to radiation compared with non‐Asian patients [22]. Second, the assessment of ILD was based on the investigators' decision, and no central review was conducted, which may have resulted in variations in the evaluation of ILD. To reduce misclassification, we provided all participating study sites with a predefined list of ILD‐related event terms to standardize case identification. However, ILD occurring after CRT may reflect combined effects of radiation and durvalumab, making it challenging to definitively diagnose radiation pneumonitis in clinical practice. Therefore, the potential misclassification of ILD assessment may not have been completely eliminated, and distinguishing the respective contributions of CRT and durvalumab to ILD development remains difficult. Third, the exclusion of certain covariates in the multivariable analysis may have affected the results. In addition, early deaths and cases of durvalumab discontinuation may represent competing risks for ILD. More than 95% of the ILD cases observed in this study occurred within 6 months after the initiation of durvalumab, and only 3.5% of the patients died within that 6‐month period (data not shown). Furthermore, the median duration of durvalumab treatment was 258 days (approximately 8 months), an appropriate length of time for assessing ILD risk. However, there were a certain number of early discontinuations, and the impact on the analysis results cannot be ruled out. Another limitation of this study is that not all patients who received durvalumab in clinical settings were enrolled in this study, potentially introducing selection bias regarding participating sites and patients. Finally, because the cutoff value for V5 is inconsistent across previous studies, the present study adopted the median value as the cutoff, which was prespecified in the statistical analysis plan. While this enabled the assessment of this variable for importance, the generalizability of the results is limited.

Despite these limitations, this study provides valuable insights into real‐world ILD management during and after durvalumab treatment. To the best of our knowledge, AYAME represents the largest prospective observational study of durvalumab in a real‐world setting conducted to date. The evidence from this analysis should help establish effective management and prevention strategies to reduce the risk of ILD occurrence in patients treated with durvalumab in clinical practice. These findings may also facilitate better decision‐making and patient care related to the PACIFIC regimen, ultimately aiming to improve patient outcomes by decreasing the risk of ILD.

In conclusion, this analysis of the AYAME study represents the first comprehensive evaluation of long‐term safety of durvalumab with a focus on ILD in Japanese patients with stage III unresectable NSCLC. Our findings showed that V20 ≥ 25%, V5 ≥ median (37.85%), and presence of grade 1 ILD before durvalumab administration were associated with the incidence of grade ≥ 2 ILD. A substantial proportion of patients were able to restart durvalumab after ILD‐related interruption, and ILD incidence during subsequent treatment was modest when IO drugs were not used. These findings may contribute to establishing therapeutic and prophylactic strategies to minimize the risk of ILD in patients treated with the PACIFIC regimen in clinical practice.

## Author Contributions

**Shinya Uematsu:** methodology, project administration, writing – original draft, writing – review and editing. **Nobuyuki Yamamoto:** conceptualization, investigation, methodology, project administration, resources, supervision, writing – review and editing. **Kiichiro Ninomiya:** conceptualization, investigation, methodology, writing – review and editing, project administration, supervision, resources. **Chikako Iwao:** conceptualization, methodology, project administration, supervision, writing – original draft, writing – review and editing. **Masakazu Fujiwara:** methodology, project administration, writing – original draft, writing – review and editing. **Burak Akdemir:** methodology, project administration, writing – original draft, writing – review and editing. **Ayako Fukui:** methodology, project administration, supervision, writing – original draft, writing – review and editing. **Ichiro Yoshino:** conceptualization, investigation, methodology, project administration, resources, supervision, writing – review and editing. **Hiroshi Kitagawa:** methodology, project administration, supervision, writing – original draft, writing – review and editing. **Ryo Koto:** methodology, project administration, writing – original draft, writing – review and editing. **Hirotsugu Kenmotsu:** conceptualization, investigation, methodology, writing – review and editing, project administration, supervision, resources. **Akihiko Gemma:** conceptualization, investigation, methodology, writing – review and editing, project administration, supervision, resources. **Tetsuya Mitsudomi:** conceptualization, investigation, methodology, writing – review and editing, project administration, supervision, resources. **Yoshinobu Saito:** conceptualization, investigation, methodology, writing – review and editing, project administration, supervision, resources.

## Funding

This work was supported by AstraZeneca K.K.

## Conflicts of Interest

Nobuyuki Yamamoto has received support for this manuscript from AstraZeneca K.K.; grants/contracts from PRiME‐R Inc., Amgen K.K., EPS Co. Ltd., Ono Pharmaceutical Co. Ltd., GlaxoSmithKline K.K., Chugai Pharmaceutical Co. Ltd., Eli Lilly Japan K.K., Novartis Japan., Pfizer R&D Japan, Medpace Japan K.K., Janssen Pharmaceutical K.K., IQVIA Solutions Japan G.K., AstraZeneca K.K., AbbVie Inc., A2 Healthcare Corporation, Kyowa Kirin Co. Ltd., Taiho Pharmaceutical Co. Ltd., Syneos Health Clinical K.K., Nippon Boehringer Ingelheim Co. Ltd., Nippon Chemical Industrial Co. Ltd., Bristol Myers Squibb, Medical Market Vision Co. Ltd., RPM Co. Ltd., and Merck Sharp & Dohme; and honoraria from AstraZeneca K.K., Accuray Japan K.K., Otsuka Pharmaceutical Co. Ltd., Guardant Health Japan Corp., Kyorin Pharmaceutical Co. Ltd., Daiichi Sankyo Inc., Takeda Pharmaceutical Co. Ltd., Tsumura & Co., Eli Lilly Japan K.K., Novartis Japan, NovoCure Ltd., Miyarisan Pharmaceutical Co. Ltd., MedPeer Inc., USACO Corporation, Amgen K.K., AbbVie Inc., Ono Pharmaceutical Co. Ltd., Kyowa Kirin Co. Ltd., GlaxoSmithKline K.K., Taiho Pharmaceutical Co. Ltd., Chugai Pharmaceutical Co. Ltd., Terumo.co.jp., Nippon Boehringer Ingelheim Co. Ltd., Nippon Chemical Industrial Co. Ltd., Pfizer Japan, Merck Biopharma Co. Ltd., Janssen Pharmaceutical K.K., and Merck Sharp & Dohme. Hirotsugu Kenmotsu has received support for this manuscript from AstraZeneca K.K.; grants/contracts from Ono Pharmaceutical Co. Ltd., Novartis Pharma K.K., Eli Lilly K.K., AstraZeneca K.K., and Loxo Oncology; and honoraria from Amgen Inc., AstraZeneca K.K., Bayer, Boehringer Ingelheim, Bristol‐Myers Squibb, Chugai Pharmaceutical Co. Ltd., Daiichi Sankyo Co. Ltd., Merck, MSD, Novartis Pharma K.K., Ono Pharmaceutical Co. Ltd., Pfizer, Taiho Pharma, Takeda Pharmaceutical Co. Ltd., Janssen Pharmaceutical K.K., Thermo Fisher Scientific Inc., and Guardant. Kiichiro Ninomiya has received honoraria from AstraZeneca, Boehringer Ingelheim, Kyowa Kirin, Lilly Japan, Chugai Pharmaceutical Co. Ltd., Nippon Kayaku, Taiho Pharmaceutical Co. Ltd., MSD K.K., Ono Pharmaceutical Co. Ltd., Takeda Pharmaceutical Co. Ltd., Pfizer Inc., Bristol Myers Squibb, Elekta K.K., Janssen Pharma, Daiichi Sankyo, Amgen K.K., Novartis, and Guardant Health Japan Corp. Burak Akdemir, Shinya Uematsu, Ayako Fukui, Ryo Koto, Masakazu Fujiwara, Chikako Iwao, and Hiroshi Kitagawa are employees of AstraZeneca K.K. Ichiro Yoshino has received honoraria from AstraZeneca, Covidien Japan, Takeda Pharmaceutical Co. Ltd., Chugai Pharmaceutical Co. Ltd., and Johnson & Johnson. Akihiko Gemma has received honoraria from Daiichi Sankyo and Nippon Kayaku. Tetsuya Mitsudomi has received support for this manuscript from AstraZeneca K.K.; and honoraria from AstraZeneca. Yoshinobu Saito has received honoraria for lectures from AstraZeneca K.K., MSD K.K., and Bristol‐Myers Squibb K.K.

## Supporting information


**Table S1:** Baseline characteristics in the safety analysis population.
**Table S2:** ILD frequency by MedDRA PT in the safety analysis population.
**Table S3:** Intervention for ILD categorized by measures taken for durvalumab in patients who developed ILD in the safety analysis population.
**Table S4:** Intervention for ILD categorized by presence or absence of re‐start of durvalumab treatment in patients with durvalumab treatment interruption (*n* = 111) in the safety analysis population.
**Table S5:** ILD in subgroups categorized by V20 in the safety analysis population.
**Table S6:** ILD in subgroups categorized by V5 in the safety analysis population.
**Table S7:** ILD in subgroups categorized by type of irradiation in the safety analysis population.
**Figure S1:** Analysis population.
**Figure S2:** Histogram of time to ILD onset after the start of first subsequent treatment by first subsequent treatment regimen in the safety analysis population by (A) EGFR‐TKI, (B) other TKI, (C) IO alone, (D) chemotherapy/other, and (E) IO + chemotherapy/other.

## Data Availability

The data that support the findings of this study are available from the corresponding author upon reasonable request.

## References

[1] F. Bray , M. Laversanne , H. Sung , et al., “Global Cancer Statistics 2022: GLOBOCAN Estimates of Incidence and Mortality Worldwide for 36 Cancers in 185 Countries,” CA: A Cancer Journal for Clinicians 74 (2024): 229–263. 38572751 10.3322/caac.21834

[2] H. Horinouchi , M. Kusumoto , Y. Yatabe , K. Aokage , S. I. Watanabe , and S. Ishikura , “Lung Cancer in Japan,” Journal of Thoracic Oncology 17 (2022): 353–361. 35216731 10.1016/j.jtho.2021.11.020

[3] D. Planchard , S. Popat , K. Kerr , et al., “Metastatic Non‐Small Cell Lung Cancer: ESMO Clinical Practice Guidelines for Diagnosis, Treatment and Follow‐Up,” Annals of Oncology 29 (2018): iv192–iv237. 30285222 10.1093/annonc/mdy275

[4] A. Casal‐Mouriño , A. Ruano‐Ravina , M. Lorenzo‐González , et al., “Epidemiology of Stage III Lung Cancer: Frequency, Diagnostic Characteristics, and Survival,” Translational Lung Cancer Research 10 (2021): 506–518. 33569332 10.21037/tlcr.2020.03.40 PMC7867742

[5] S. J. Antonia , A. Villegas , D. Daniel , et al., “Durvalumab After Chemoradiotherapy in Stage III Non‐Small‐Cell Lung Cancer,” New England Journal of Medicine 377 (2017): 1919–1929. 28885881 10.1056/NEJMoa1709937

[6] S. J. Antonia , A. Villegas , D. Daniel , et al., “Overall Survival With Durvalumab After Chemoradiotherapy in Stage III NSCLC,” New England Journal of Medicine 379 (2018): 2342–2350. 30280658 10.1056/NEJMoa1809697

[7] D. R. Spigel , C. Faivre‐Finn , J. E. Gray , et al., “Five‐Year Survival Outcomes From the PACIFIC Trial: Durvalumab After Chemoradiotherapy in Stage III Non‐Small‐Cell Lung Cancer,” Journal of Clinical Oncology 40 (2022): 1301–1311. 35108059 10.1200/JCO.21.01308 PMC9015199

[8] M. E. Daly , N. Singh , N. Ismaila , et al., “Management of Stage III Non‐Small‐Cell Lung Cancer: ASCO Guideline,” Journal of Clinical Oncology 40 (2022): 1356–1384. 34936470 10.1200/JCO.21.02528

[9] G. Pentheroudakis , “Recent eUpdate to the ESMO Clinical Practice Guidelines on Early and Locally Advanced Non‐Small‐Cell Lung Cancer (NSCLC),” Annals of Oncology 31 (2020): 1265–1266. 32502714 10.1016/j.annonc.2020.05.023

[10] H. Kenmotsu , Y. Saito , K. Ninomiya , et al., “Long‐Term Safety and Effectiveness of Durvalumab in Unresectable Stage III Non‐Small Cell Lung Cancer in Japan: A Multicenter Prospective Study (AYAME),” Journal of Thoracic Oncology 21, no. 1 (2025): 160–173. 40835220 10.1016/j.jtho.2025.08.010

[11] M. A. Couey , R. B. Bell , A. A. Patel , et al., “Delayed Immune‐Related Events (DIRE) After Discontinuation of Immunotherapy: Diagnostic Hazard of Autoimmunity at a Distance,” Journal for Immunotherapy of Cancer 7 (2019): 165. 31269983 10.1186/s40425-019-0645-6 PMC6609357

[12] Pharmaceutical and Medical Devices Agency , “Report on the Deliberation Results,” Durvalumab (2018), https://www.pmda.go.jp/files/000238551.pdf.

[13] H. Murakami , H. Horinouchi , H. Harada , et al., “Deciphering the Clinical Features of Heterogeneous Stage III Non‐Small Cell Lung Cancer in Japanese Real‐World Clinical Practice: Expanded Cohort of the SOLUTION Study,” Lung Cancer 165 (2022): 152–163. 35144145 10.1016/j.lungcan.2021.12.005

[14] S. Murata , H. Horinouchi , M. Morishita , et al., “Impact of Durvalumab on the Duration and Complexity of Corticosteroid Therapy for Pneumonitis After Chemoradiotherapy,” Clinical Lung Cancer 25 (2024): e369–378.e3. 39079873 10.1016/j.cllc.2024.06.009

[15] G. Saito , Y. Oya , Y. Taniguchi , et al., “Real‐World Survey of Pneumonitis and Its Impact on Durvalumab Consolidation Therapy in Patients With Non‐Small Cell Lung Cancer Who Received Chemoradiotherapy After Durvalumab Approval (HOPE‐005/CRIMSON),” Lung Cancer 161 (2021): 86–93. 34543942 10.1016/j.lungcan.2021.08.019

[16] N. Girard , J. Bar , P. Garrido , et al., “Treatment Characteristics and Real‐World Progression‐Free Survival in Patients With Unresectable Stage III NSCLC Who Received Durvalumab After Chemoradiotherapy: Findings From the PACIFIC‐R Study,” Journal of Thoracic Oncology 18 (2023): 181–193. 36307040 10.1016/j.jtho.2022.10.003

[17] Y. Tsukita , T. Yamamoto , H. Mayahara , et al., “Intensity‐Modulated Radiation Therapy With Concurrent Chemotherapy Followed by Durvalumab for Stage III Non‐Small Cell Lung Cancer: A Multi‐Center Retrospective Study,” Radiotherapy and Oncology 160 (2021): 266–272. 34023330 10.1016/j.radonc.2021.05.016

[18] K. Suresh , K. R. Voong , B. Shankar , et al., “Pneumonitis in Non‐Small Cell Lung Cancer Patients Receiving Immune Checkpoint Immunotherapy: Incidence and Risk Factors,” Journal of Thoracic Oncology 13 (2018): 1930–1939. 30267842 10.1016/j.jtho.2018.08.2035

[19] T. Seki , Y. Nishino , F. Tanji , et al., “Cigarette Smoking and Lung Cancer Risk According to Histologic Type in Japanese Men and Women,” Cancer Science 104 (2013): 1515–1522. 23992614 10.1111/cas.12273 PMC7656551

[20] T. Hasegawa , R. Ariyasu , H. Tanaka , et al., “Subsequent Treatment for Locally Advanced Non‐Small‐Cell Lung Cancer That Progressed After Definitive Chemoradiotherapy and Consolidation Therapy With Durvalumab: A Multicenter Retrospective Analysis (TOPGAN 2021‐02),” Cancer Chemotherapy and Pharmacology 92 (2023): 29–37. 37243795 10.1007/s00280-023-04547-2

[21] A. J. Schoenfeld , K. C. Arbour , H. Rizvi , et al., “Severe Immune‐Related Adverse Events Are Common With Sequential PD‐(L)1 Blockade and Osimertinib,” Annals of Oncology 30 (2019): 839–844. 30847464 10.1093/annonc/mdz077 PMC7360149

[22] T. Liu , S. Li , S. Ding , et al., “Comparison of Post‐Chemoradiotherapy Pneumonitis Between Asian and Non‐Asian Patients With Locally Advanced Non‐Small Cell Lung Cancer: A Systematic Review and Meta‐Analysis,” EClinicalMedicine 64 (2023): 102246. 37781162 10.1016/j.eclinm.2023.102246 PMC10539643

