# Supplementary material for: Interstitial Lung Disease in Patients With Unresectable Stage III NSCLC Treated With Chemoradiotherapy Followed by Durvalumab in Japan: Analysis From the Multicenter Prospective AYAME Study
Source: Thorac Cancer. 2026 May 19;17(10):e70299. doi: 10.1111/1759-7714.70299 (PMC13184635; doi:10.1111/1759-7714.70299)
Supplement: Supplementary file 1 — Table S1: Baseline characteristics in the safety analysis population. Table S2: ILD frequency by MedDRA PT in the safety analysis population. Table S3: Intervention for ILD categorized by measures taken for durvalumab in patients who developed ILD in the safety analysis population. Table S4: Intervention for ILD categorized by presence or absence of re‐start of durvalumab treatment in patients with durvalumab treatment interruption (n = 111) in the safety analysis population. Table S5: ILD in subgroups categorized by V20 in the safety analysis population. Table S6: ILD in subgroups categorized by V5 in the safety analysis population. Table S7: ILD in subgroups categorized by type of irradiation in the safety analysis population. Figure S1: Analysis population. Figure S2: Histogram of time to ILD onset after the start of first subsequent treatment by first subsequent treatment regimen in the safety analysis population by (A) EGFR‐TKI, (B) other TKI, (C) IO alone, (D) chemotherapy/other, and (E) IO + chemotherapy/other. [file TCA-17-e70299-s001.docx]

**Interstitial lung disease in patients with unresectable stage III NSCLC treated with chemoradiotherapy followed by durvalumab in Japan: analysis from the multicenter prospective AYAME study**

**Authors:** Nobuyuki YAMAMOTO, Hirotsugu KENMOTSU, Kiichiro NINOMIYA, Burak AKDEMIR, Shinya UEMATSU, Ayako FUKUI, Ryo KOTO, Masakazu FUJIWARA, Chikako IWAO, Hiroshi KITAGAWA, Ichiro YOSHINO, Akihiko GEMMA, Tetsuya MITSUDOMI, Yoshinobu SAITO

**Corresponding author:** Nobuyuki Yamamoto, Internal Medicine III, Wakayama Medical University, 811-1 Kimiidera, Wakayama 641-8509, Japan. Tel: 073-441-0619; Fax: 073-446-2877; Email: [nbyamamo@wakayama-med.ac.jp](mailto:nbyamamo@wakayama-med.ac.jp)

**Supporting information**

## **TABLE S1**

Baseline characteristics in the safety analysis population.

| Characteristics | n=511 |
| --- | --- |
| Age, median (range), years | 69.0 (31.0–88.0) |
| Age category, n (%) |  |
| ≥20 to <75 years | 392 (76.7) |
| ≥75 years | 119 (23.3) |
| Sex, n (%) |  |
| Male | 393 (76.9) |
| Female | 118 (23.1) |
| Smoking status, n (%) |  |
| Current smoker | 71 (13.9) |
| Former smoker | 380 (74.4) |
| Never | 60 (11.7) |
| ECOG PS before the start of durvalumab, n (%) |  |
| 0 | 262 (51.3) |
| 1 | 239 (46.8) |
| 2 | 7 (1.4) |
| 3 | 1 (0.2) |
| 4 | 0 |
| Missing | 2 (0.4) |
| Disease stage, n (%) |  |
| IIIA | 205 (40.1) |
| IIIB | 195 (38.2) |
| IIIC | 62 (12.1) |
| Postoperative recurrence [equivalent to III] | 45 (8.8) |
| Missing | 4 (0.8) |
| Histology, n (%) |  |
| Adenocarcinoma | 254 (49.7) |
| Squamous cell carcinoma | 208 (40.7) |
| Neuroendocrine tumor [non-small cell cancer] | 12 (2.3) |
| Other | 37 (7.2) |
| Presence or absence of concurrent diseases, n (%) |  |
| Presence | 377 (73.8) |
| Absence | 134 (26.2) |
| Details of concurrent diseases,^†^ n (%) |  |
| ILD [including radiation pneumonitis, G1] | 54 (10.6) |
| ILD [including radiation pneumonitis, ≥G2] | 2 (0.4) |
| COPD | 91 (17.8) |
| Other | 339 (66.3) |
| Presence or absence of ILD and its severity, n (%) |  |
| Presence [G1] | 54 (10.6) |
| Presence [≥G2] | 2 (0.4) |
| Absence | 455 (89.0) |
| Presence or absence of autoimmune diseases,^†^ n (%) |  |
| Presence | 22 (4.3) |
| Absence | 489 (95.7) |
| Chemotherapy regimen,^‡^ n (%) |  |
| CBDCA+PTX | 225 (44.0) |
| CDDP+VNR | 108 (21.1) |
| CDDP+DTX | 35 (6.8) |
| CBDCA | 51 (10.0) |
| CDDP+S-1 | 62 (12.1) |
| Other | 30 (5.9) |
| Type of irradiation,^§^ n (%) |  |
| Photon therapy | 507 (99.2) |
| Conventional (2D) | 3 (0.6) |
| 3D-CRT | 358 (70.6) |
| IMRT | 160 (31.6) |
| SRT | 2 (0.4) |
| Total radiation dose (actual irradiated dose), median (range), Gy^¶^ | 60.0 (40.0–102.0) |
| <54 | 3 (0.6) |
| ≥54 to ≤66 | 502 (98.2) |
| >66 | 6 (1.2) |
| MLD, median (range), Gy^\|\|^ (n=479) | 11.9 (0.9–60.0) |
| <13 | 280 (54.8) |
| ≥13 | 199 (38.9) |
| V20 (%), median (range)^\|\|^ (n=479) | 20.9 (2.3–39.0) |
| <25 | 323 (63.2) |
| ≥25 to <30 | 101 (19.8) |
| ≥30 to <35 | 46 (9.0) |
| ≥35 | 9 (1.8) |
| V5 (%), median (range)^\|\|^ (n=478) | 37.9 (3.5–89.4) |
| <27 | 117 (22.9) |
| ≥27 to <37 | 112 (21.9) |
| ≥37 to <47 | 126 (24.7) |
| ≥47 | 123 (24.1) |

^†^Patients with >1 concurrent disease/autoimmune disease were counted for each concurrent disease/autoimmune disease (duplicate counting). Autoimmune diseases included hypothyroidism, rheumatoid arthritis, autoimmune thyroiditis, alopecia areata, hyperthyroidism, scleritis, scleroderma, chronic thyroiditis, Cronkhite-Canada syndrome, microscopic polyangiitis, type 1 diabetes mellitus, and immunoglobulin G4–related disease.
^‡^The first regimen was counted if multiple regimens were present.
^§^Patients who received >1 type of radiation therapy were counted for each radiation therapy (duplicate counting).
^¶^For patients who received >1 kind of radiation therapy, values were summed so that one datum per patient was used for the calculation.
^||^For patients who received >1 type of radiation therapy with >1 value (other than 0) available per patient, data from such patients were handled as “missing” because MLD, V20, and V5 could not be summed.
2D, two-dimensional; 3D-CRT, three-dimensional conformal radiation therapy; CBDCA, carboplatin; CBDCA+PTX, carboplatin + paclitaxel; CDDP+DTX, cisplatin + docetaxel; CDDP+S-1, cisplatin + S-1; CDDP+VNR, cisplatin + vinorelbine; COPD, chronic obstructive pulmonary disease; ECOG PS, Eastern Cooperative Oncology Group performance status; G, grade; ILD, interstitial lung disease; IMRT, intensity-modulated radiation therapy; MLD, mean lung dose; SRT, stereotactic radiation therapy; V20, volume of lung parenchyma that received 20 Gy; V5, volume of lung parenchyma that received 5 Gy.

## **TABLE S2**

ILD frequency by MedDRA PT in the safety analysis population.

|  | **n (% of total N=511)** | **Events^§^ n (%)** |
| --- | --- | --- |
| ILD | 383 (75.0) | 433 |
| Radiation pneumonitis | 319 (62.4) | 333 (76.9) |
| Interstitial lung disease | 42 (8.2) | 45 (10.4) |
| Pneumonitis | 46 (9.0) | 48 (11.1) |
| Organizing pneumonia | 5 (1.0) | 5 (1.2) |
| Radiation fibrosis – lung | 2 (0.4) | 2 (0.5) |

^§^Events were counted in duplicate.
ILD, interstitial lung disease; MedDRA, Medical Dictionary for Regulatory Activities; PT, preferred term.

**TABLE S3** Intervention for ILD categorized by measures taken for durvalumab in patients who developed ILD in the safety analysis population.

| **Maximum ILD grade** | **n=383** | | | | |
| --- | --- | --- | --- | --- | --- |
|  | **No change (n=118), n (%)** | **Dose reduction (n=0), n (%)** | **Permanent discontinuation (n=121), n (%)** | **Dose interruption (n=111), n (%)** | **Not applicable**^†^ **(n=33), n (%)** |
| 1 | 109 (92.4) | 0 | 16 (13.2) | 47 (42.3) | 17 (51.5) |
| 2 | 8 (6.8) | 0 | 63 (52.1) | 57 (51.4) | 12 (36.4) |
| 3 | 1 (0.8) | 0 | 38 (31.4) | 7 (6.3) | 4 (12.1) |
| 4 | 0 | 0 | 0 | 0 | 0 |
| 5 | 0 | 0 | 4 (3.3) | 0 | 0 |

ILD events were counted from the start of durvalumab treatment until the initiation of subsequent treatment. For patients who experienced more than one ILD event, a single representative ILD event was selected per analysis using a predefined priority (i.e., intervention for ILD). Therefore, the total number of patients by grade may differ from those presented in Table 1.
^†^Not applicable included cases where durvalumab was not the suspected drug and cases where durvalumab was the suspected drug but durvalumab treatment was already discontinued before the onset of ILD.

ILD, interstitial lung disease.

## **TABLE S4**

Intervention for ILD categorized by presence or absence of re-start of durvalumab treatment in patients with durvalumab treatment interruption (n=111) in the safety analysis population.

| **Maximum ILD grade** | **Intervention with steroid therapy** | **Re-start of durvalumab treatment** | |
| --- | --- | --- | --- |
|  |  | **Presence (n=99)** | **Absence (n=12)** |
|  |  | **n (%)** | **n (%)** |
| 1 | Present | 3 (3.0) | 0 |
|  | Absent | 43 (43.4) | 1 (8.3) |
| 2 | Present | 32 (32.3) | 9 (75.0) |
|  | Absent | 14 (14.1) | 2 (16.7) |
| 3 | Present | 5 (5.1) | 0 |
|  | Absent | 2 (2.0) | 0 |
| 4 | Present | 0 | 0 |
|  | Absent | 0 | 0 |
| 5 | Present | 0 | 0 |
|  | Absent | 0 | 0 |

ILD events were counted from the start of durvalumab treatment until the initiation of subsequent treatment.

ILD, interstitial lung disease.

## **TABLE S5**

ILD in subgroups categorized by V20 in the safety analysis population.

|  | **V20 (%)** | | | |
| --- | --- | --- | --- | --- |
|  | **<25 n=323** | **≥25 to <30 n=101** | **≥30 to <35 n=46** | **≥35 n=9** |
| Presence or absence of ILD onset, n (%) | | | | |
| Present | 228 (70.6) | 87 (86.1) | 43 (93.5) | 8 (88.9) |
| Absent | 95 (29.4) | 14 (13.9) | 3 (6.5) | 1 (11.1) |
| Number of ILD events, n (%) | | | | |
| 0 | 95 (29.4) | 14 (13.9) | 3 (6.5) | 1 (11.1) |
| 1 | 202 (62.5) | 74 (73.3) | 39 (84.8) | 8 (88.9) |
| 2 | 23 (7.1) | 12 (11.9) | 4 (8.7) | 0 |
| ≥3 | 3 (0.9) | 1 (1.0) | 0 | 0 |
| Maximum ILD grade, n (%) | | | | |
| Any | 228 (70.6) | 87 (86.1) | 43 (93.5) | 8 (88.9) |
| 1 | 133 (41.2) | 28 (27.7) | 15 (32.6) | 1 (11.1) |
| 2 | 73 (22.6) | 44 (43.6) | 18 (39.1) | 4 (44.4) |
| 3 | 20 (6.2) | 14 (13.9) | 9 (19.6) | 3 (33.3) |
| 4 | 0 | 0 | 0 | 0 |
| 5 | 2 (0.6) | 1 (1.0) | 1 (2.2) | 0 |

ILD events were counted from the start of durvalumab treatment until the initiation of subsequent treatment.
ILD, interstitial lung disease; V20, volume of lung parenchyma that received 20 Gy.

## **TABLE S6**

ILD in subgroups categorized by V5 in the safety analysis population.

|  | **V5** | |
| --- | --- | --- |
|  | **<Median**^†^ **n=239** | **≥Median**^†^ **n=239** |
| Presence or absence of ILD onset, n (%) | | |
| Present | 159 (66.5) | 206 (86.2) |
| Absent | 80 (33.5) | 33 (13.8) |
| Number of ILD events, n (%) | | |
| 0 | 80 (33.5) | 33 (13.8) |
| 1 | 144 (60.3) | 178 (74.5) |
| 2 | 15 (6.3) | 24 (10.0) |
| ≥3 | 0 | 4 (1.7) |
| Maximum ILD grade, n (%) | | |
| Any | 159 (66.5) | 206 (86.2) |
| 1 | 94 (39.3) | 83 (34.7) |
| 2 | 50 (20.9) | 89 (37.2) |
| 3 | 14 (5.9) | 31 (13.0) |
| 4 | 0 | 0 |
| 5 | 1 (0.4) | 3 (1.3) |

ILD events were counted from the start of durvalumab treatment until the initiation of subsequent treatment.
^†^Median = 37.85%.
ILD, interstitial lung disease; V5, volume of lung parenchyma that received 5 Gy.

## **TABLE S7**

ILD in subgroups categorized by type of irradiation in the safety analysis population.

|  | **Irradiation method**^†^ | | | |
| --- | --- | --- | --- | --- |
|  | **Conventional (2D) n=3** | **3D-CRT n=358** | **IMRT n=160** | **SRT n=2** |
| Presence or absence of ILD onset, n (%) | | | | |
| Present | 3 (100.0) | 263 (73.5) | 122 (76.3) | 2 (100.0) |
| Absent | 0 | 95 (26.5) | 38 (23.8) | 0 |
| Number of ILD events, n (%) | | | | |
| 0 | 0 | 95 (26.5) | 38 (23.8) | 0 |
| 1 | 3 (100.0) | 238 (66.5) | 102 (63.8) | 1 (50.0) |
| 2 | 0 | 24 (6.7) | 17 (10.6) | 1 (50.0) |
| ≥3 | 0 | 1 (0.3) | 3 (1.9) | 0 |
| Maximum ILD grade, n (%) | | | | |
| Any | 3 (100.0) | 263 (73.5) | 122 (76.3) | 2 (100.0) |
| 1 | 1 (33.3) | 128 (35.8) | 55 (34.4) | 0 |
| 2 | 2 (66.7) | 91 (25.4) | 50 (31.3) | 1 (50.0) |
| 3 | 0 | 40 (11.2) | 17 (10.6) | 0 |
| 4 | 0 | 0 | 0 | 0 |
| 5 | 0 | 4 (1.1) | 0 | 1 (50.0) |

ILD events were counted from the start of durvalumab treatment until the initiation of subsequent treatment.
^†^Patients who received more than one type of radiation therapy were counted for each radiation therapy (duplicate counting).
2D, two-dimensional; 3D-CRT, three-dimensional conformal radiation therapy; ILD, interstitial lung disease; IMRT, intensity-modulated radiation therapy; SRT, stereotactic radiation therapy.

## **FIGURE S1**

Analysis population.


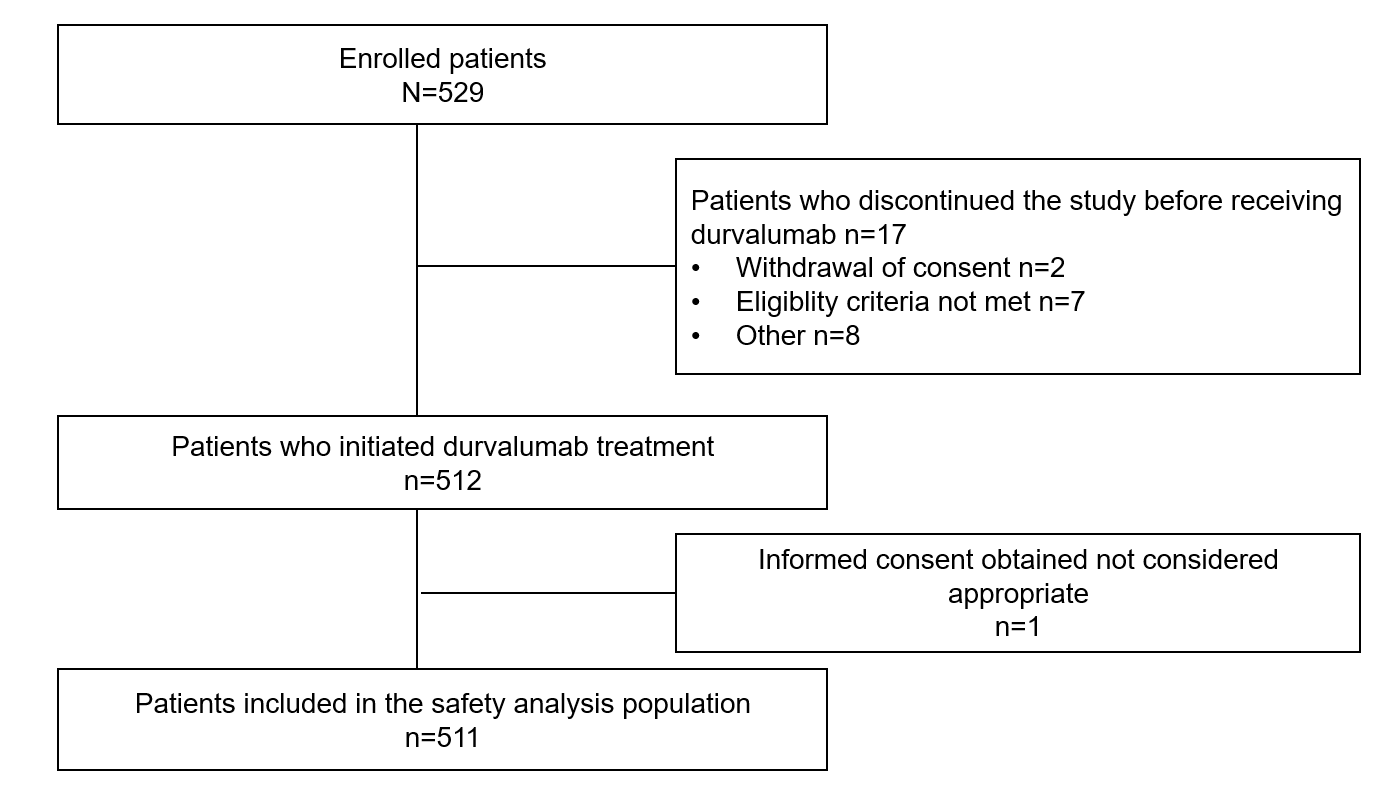


## **FIGURE S2**

Histogram of time to ILD onset after the start of first subsequent treatment by first subsequent treatment regimen in the safety analysis population by A) EGFR-TKI, B) other TKI, C) IO alone, D) chemotherapy/other, and E) IO + chemotherapy/other.


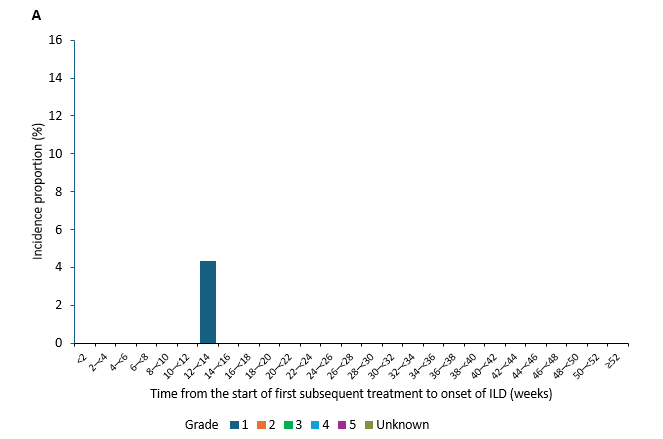

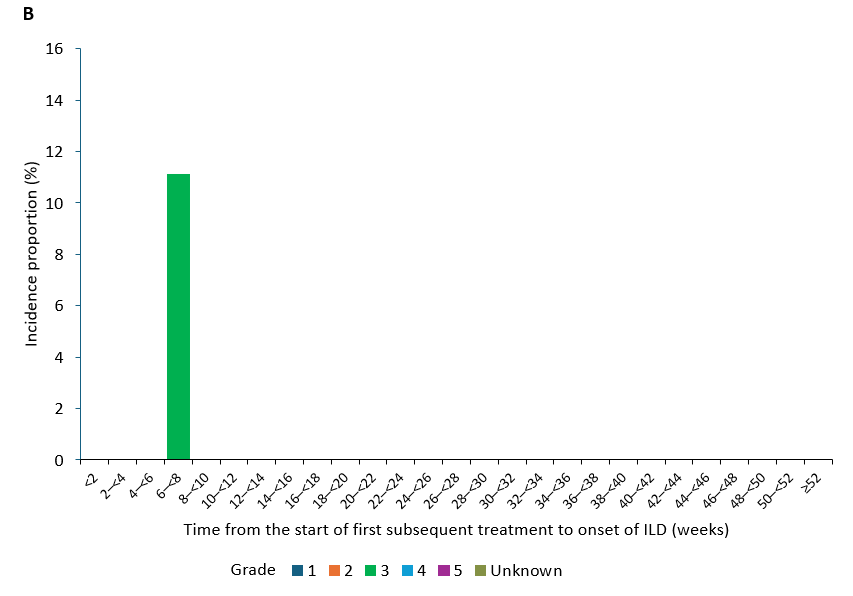

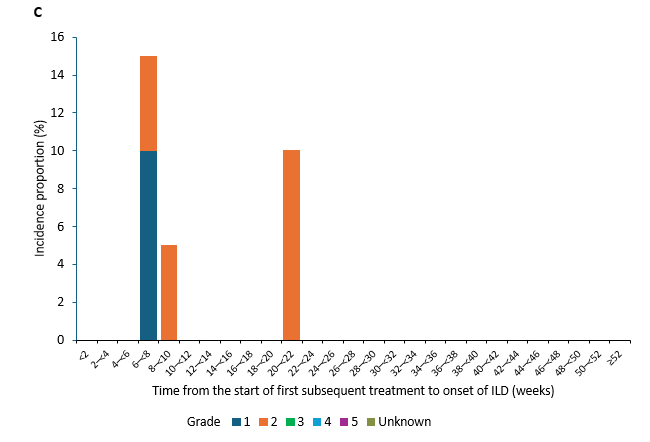

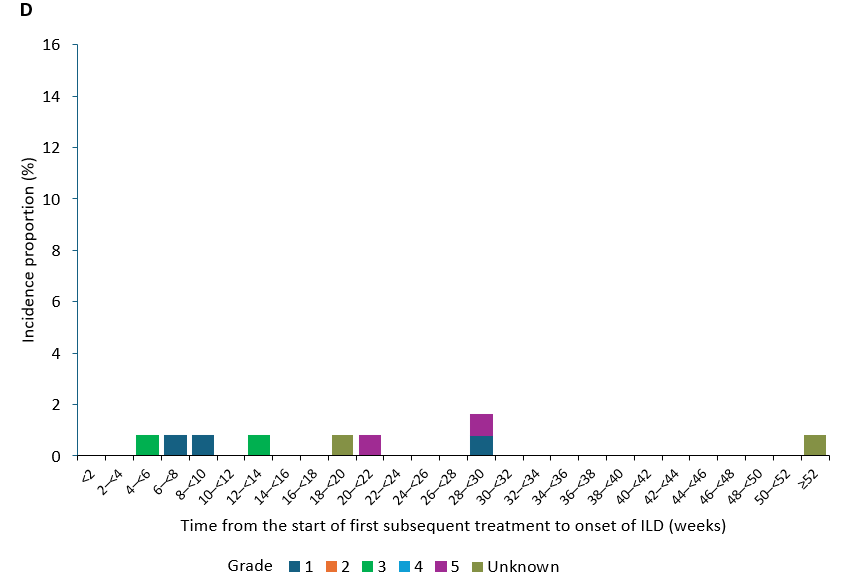

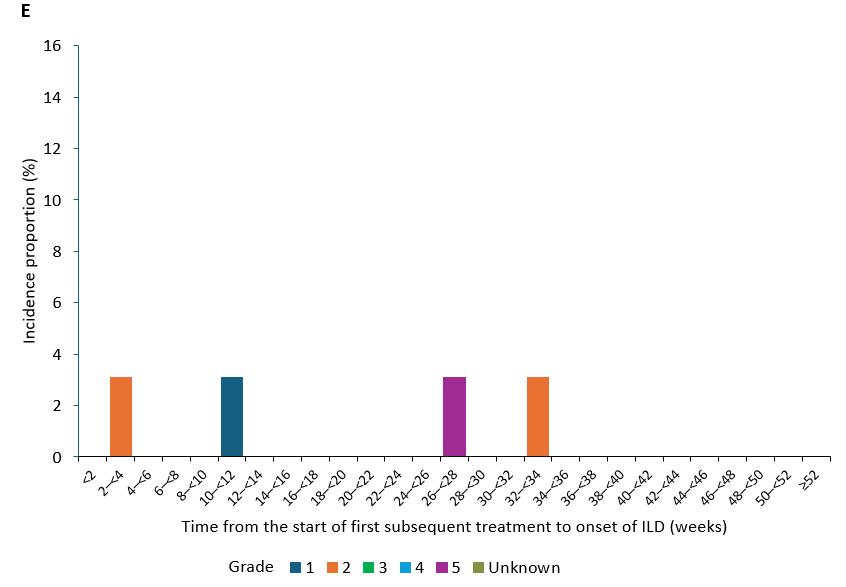

EGFR-TKI: gefitinib, erlotinib, afatinib, osimertinib, and dacomitinib. Other TKI: crizotinib, alectinib.
IO: nivolumab ± ipilimumab, pembrolizumab, atezolizumab. Chemo (cytotoxic anticancer drugs)/other: any drugs other than those listed as EGFR-TKI, other TKI, and IO. IO + chemo/other: a combination of drugs listed as IO and chemo/other.
EGFR-TKI; epidermal growth factor receptor-tyrosine kinase inhibitor; ILD, interstitial lung disease; IO, immune-oncology; TKI, tyrosine kinase inhibitor.
